# Delirium is under-reported in discharge summaries and in hospital administrative systems: a systematic review

**DOI:** 10.56392/001c.74541

**Published:** 2023-05-15

**Authors:** Temi Ibitoye, Sabrina So, Susan D. Shenkin, Atul Anand, Matthew J. Reed, Emma R.L.C. Vardy, Sarah T Pendelbury, Alasdair M.J. MacLullich

**Affiliations:** 1 Edinburgh Delirium Research Group, Ageing and Health, Usher Institute, University of Edinburgh; 2 Edinburgh Royal Infirmary, NHS Lothian; 3 Edinburgh Delirium Group, Ageing and Health, Usher Institute, University of Edinburgh; 4 Advanced Care Research Centre, Usher Institute, University of Edinburgh; 5 Centre for Cardiovascular Science, University of Edinburgh; 6 Acute Care Edinburgh, Usher Institute, University of Edinburgh; 7 Salford Care Organisation, Northern Care Alliance NHS Foundation Trust; 8 NIHR Applied Research Collaboration Greater Manchester, University of Manchester; 9 Manchester Academic Health Science Centre, School of Health Sciences, University of Manchester; 10 NIHR Oxford Biomedical Research Centre, Oxford University Hospitals NHS Trust; 11 Acute (Internal) Medicine and Geratology, Oxford University Hospitals NHS Trust; 12 Wolfson Centre for Prevention of Stroke and Dementia Nuffield Department of Clinical Neurosciences, The University of Oxford

**Keywords:** delirium, discharge summaries, geriatric, healthcare coding

## Abstract

**Background:**

Accurate recording of delirium in discharge summaries (DS) and hospital administrative systems (HAS) is critical for patient care.

**Objective:**

To systematically review studies reporting the frequency of delirium documentation and coding in DS and HAS, respectively.

**Method:**

We searched Medline, Embase, PsycINFO and Web of Science databases from inception to 23 June 2021. Eligibility criteria included requiring the term *delirium* in DS or HAS. Screening and full-text reviews were performed independently by two reviewers. Risk of bias (RoB) was assessed using the Effective Public Health Practice Project tool.

**Results:**

The search yielded 7,910 results; 24 studies were included. The studies were heterogeneous in design and size (N=25 to 809,512). Mean age ranged from 57 to 84 years. Four studies reported only overall DS documentation and HAS coding in whole hospital or healthcare databases. Twenty studies used additional delirium ascertainment methods (e.g. chart review) in smaller patient subsets. Studies reported either DS figures only (N=8), HAS figures only (N=11), or both (N=5). Documentation rates in DS ranged from 0.1% to 64%. Coding rates in HAS ranged from 1.5% to 49%. Some studies explored the impact of race, and nurse versus physician practice. No significant differences were reported for race; one study reported that nurses showed higher documentation rates in DS relative to physicians. Most studies (N=22) had medium to high RoB.

**Conclusion:**

Delirium is a common and serious medical emergency, yet studies show considerable under-documentation and under-coding in healthcare systems. This has important implications for patient care and service planning. Healthcare systems need to take action to reach satisfactory delirium documentation and coding rates.

## Introduction

Delirium is a severe neuropsychiatric syndrome that affects 1 in 4 hospitalised older adults.^1,2^ It is associated with multiple adverse patient outcomes.^3,4^ Delirium detection is advocated in numerous guidelines and care standards for improving outcomes.^5–7^ Yet the majority of delirium remains unrecognised in most hospitals^8^ and is consequently under-documented in medical records, including discharge summaries (DS) and under-coded in hospital administrative systems (HAS).^9–11^

Hospital administrative coding translates information recorded in patient medical records to a standard coded format and is used for statistics, reimbursement and case-mix adjustments.^12^ Clinical coders rely on the accuracy of the information provided in medical records, including DS.^13^ Delirium is unlikely to be listed in HAS by clinical coders if documentation in medical records is absent or poor. This would lead to an underestimation of true delirium prevalence and incidence, lower reimbursement, and fewer resources allocated for managing delirium. DS are a form of medical records created in secondary care; they provide an overview of patient events from the point of admission up until discharge. Accurate DS are essential for high-quality communication with primary care and to inform future secondary care episodes and care pathways.^14^

Delirium documentation and coding are critical elements in providing high-quality, comprehensive delirium care, but there is little scrutiny in the academic literature. Here we report a systematic review with a narrative synthesis of published studies reporting rates of delirium documentation in DS and/or delirium coding in HAS.

## Methods

The systematic review was registered with PROSPERO on 26 February 2021 (CRD42021239547) and is reported according to PRISMA guidelines (supplementary Table 1, supplementary Figure 1).^15^

### Inclusion Criteria

Peer-reviewed studies reporting:

- hospitalised patients with diagnosed delirium, including subtype or superimposed on dementia
- documented description and/or diagnosis of delirium in DS (or equivalent), or HAS coding.
- publication in English or accessible using translation tools.

### Exclusion Criteria

We excluded studies if they:

- used only synonyms such as *confusion* or *encephalopathy* or *acute psychosis* or *altered mental status* or only reported delirium symptoms
- were in non-hospital settings, such as care homes and hospices
- were systematic reviews, meta-analyses, abstracts, letters to editors or opinion pieces.

### Search Strategy

The search strategy comprised three concepts: (1) delirium, (2) documentation or coding, and (3) DS or HAS, developed for Medline, Embase, PsycINFO and Web of Science (Supplementary Table 2). The search was performed on 13 March 2021 and updated on 23 June 2021. We used forward citation and scoped grey literature using the same concepts (Supplementary Table 3). Title, abstract and keyword screening, and full-text reviews of long-listed publications were performed independently by two reviewers (TI and SS). Conflicts were resolved by an additional reviewer (AMJM).

### Risk Of Bias

Two reviewers (TI and SS) independently assessed studies for risk of bias (RoB) using the Effective Public Health Practice Project (EPHPP) quality assessment tool.^16^ Conflicts were resolved through discussion. Studies were assessed as strong, moderate or weak for: selection bias, study design, confounding, blinding and data collection (Supplementary Table 4). We applied the global rating criteria for an overall rating. Global ratings for RoB generally ranged from moderate to high, largely due to study design, confounders and blinding (Supplementary Figure 2). Two studies had low global RoB ratings.^17,18^

### Data Extraction And Synthesis Measures

We extracted the reported delirium documentation and/or coding rates for each study in DS and HAS, respectively. Where studies used a range of codes to denote presumed delirium or synonyms (e.g. encephalopathy) but reported rates by specific code, we calculated the coding rates by delirium-specific codes only (Supplementary Table 5). Similarly, where studies did not use a diagnostic manual or coding dictionary but instead used text in the DS, we reported the documentation rates only for the specific term *delirium* rather than synonyms.

We extracted subgroup data on population characteristics (e.g. race or gender), hospital settings (e.g. geriatrics, medical, or intensive care units), structured and unstructured DS, and hospital staff (e.g. physicians or nurses).

Some studies measured delirium with additional study-specific ascertainment methods, such as chart reviews. For these, we calculated delirium *study prevalence* (total number of ascertained cases (n) in the ascertained sample (N)) (Supplementary Table 6). Among patients with study-ascertained delirium, we determined the corresponding proportion with documentation in DS or HAS.

## Results

We identified 7,910 studies, including 24 published between 1992 and 2021 (Table 1).^18–41^ One study was identified using forward citation.^41^ There was a title-abstract agreement between reviewers in 99% (Cohen’s κ 0.60) and 86% of cases (Cohen’s K 0.70) at full-text review.^42^ One article was available in Spanish^24^ and translated to English.^43^ Studies were mostly in high income counties (22), with two in Thailand and Colombia. Mean sample age ranged from 57 to 84 years; one study was in a paediatric hospital.^28^

The 24 studies were heterogeneous in design, delirium study-ascertainment method (if performed), and sample size (Tables 1−2). Studies were mainly on general medicine wards, surgical wards, or intensive care units (ICU); one was in a community hospital.^19^ Studies reported DS only (N=8), HAS only (N=11), or both (N=5). Twenty studies used additional methods to ascertain delirium rates to enable comparison with the DS and HAS figures (Table 2).

In the four studies with no additional delirium ascertainment (Table 2), samples were in entire hospital or health-care system databases (up to N=809,512). Documentation rates in DS were 0.1%^28^ and 0.9%,^27^ and delirium HAS coding rates were 1.5%,^30^ 2.9%,^17^ and 3.4%.^27^ In the 20 studies with additional delirium ascertainment (Table 2), sample sizes were smaller (between N=25 and N=1,528). Both DS documentation and HAS coding were higher in these studies: 2.9%-64% and 2.6%-49%, respectively (Supplementary Figure 2). DS and/or HAS rates were primarily reported for the population of patients with study-specific delirium ascertainment, though not exclusively.

**Figure 1:**
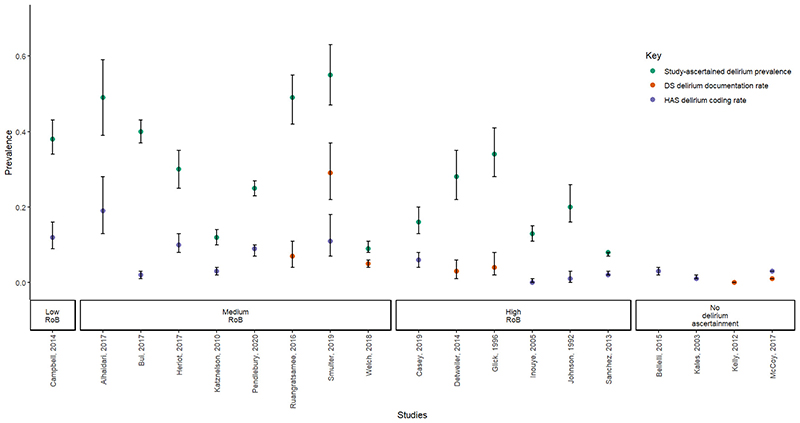
Study-ascertained delirium, DS Documentation and HAS Coding Rates ^1^ Figure 1 presents studies that reported study-ascertained delirium prevalence from a sample and reported DS documentation and/or HAS coding rates. We excluded studies where the overall sample comprised 100% delirium patients as determined by, e.g., retrospective chart review. As a result, the following studies were excluded: Chuen et al., 2021, Hope et al., 2014, van Zyl et al., 2003 and Zalon et al., 2007. We also excluded Welch et al., 2019; the authors reported that discharge documentation were available for 154 of the 222 patients identified with study-ascertained delirium. ^2^ DS documentation and/or HAS coding rates are expressed as a fraction of the overall sample size. ^3^ 95% confidence intervals are represented by the vertical black bars

Diagnosed delirium was higher than corresponding rates of DS documentation and HAS coding in studies with additional delirium ascertainment. This trended with RoB, with low and medium RoB studies reporting higher rates than high RoB studies (Figure 1).

Multiple studies used retrospective methods to determine accuracy of delirium coding.^19,32,34,36^ In a chart review of emergency admissions, Detweiler found only 9.6% of positive cases had delirium documented in their DS.^35^ Using a chart extraction tool, Hope showed 44% of study-ascertained delirium was documented in DS.^32^ Chuen found the highest rates: 64% of cases documented in DS.^36^

Among studies with prospective delirium ascertainment, Welch identified that 9.4% had delirium using DSM-IV in 1,327 acute admissions.^21^ In a study with similar methods, Welch (2019) found delirium was documented in 44 of 154 DS (29%).^20^ Ruangratsamee prospectively assessed delirium in an older acute medical population and found that delirium was documented in only 16 patient DS (15%) despite physicians having recognised 57% delirium cases.^25^ Where DS were structured, Chuen reported no differences in the odds of delirium documentation (OR 0.55, 95% CI [0.18–1.70]).^36^ However, this contrasted with a smaller study where delirium documentation was higher in structured than unstructured DS (56% v 0% respectively).^22^

A prospective study by Pendlebury reported a substantial increase had occurred from 13% in 2010 to 60% in 2018 following a system-wide multicomponent intervention consisting of audits, delirium training and educational seminars.^23^

Some studies reported on delirium DS documentation and HAS coding rates by hospital service type or hospital staff. Detweiler retrospectively compared rates of missed delirium documentation in DS; ED and Medical services had the highest rates of missed documentation (29% and 30%, respectively), followed by surgery (24%) and psychiatric services (14%).^35^ Chuen reported higher delirium DS documentation in surgical services (77%) compared with medical services (53%).^36^ One study showed DS documentation was higher for nurses (53%) than physicians (41%).^23^

Two studies reported race-disaggregated HAS delirium coding. One found no difference in between African-Americans and non-African-Americans,^18^ contrasting with the other reporting substantially lower coding in African-American patients compared with Caucasian patients (15% vs 78%).^30^

## Discussion

We identified 24 published studies reporting delirium documentation in DS or coding in HAS. Whole-system studies without additional ascertainment reported delirium documentation and/or coding rates that were far lower than expected rates. Documentation and coding rates were much higher where there was a dedicated component of delirium ascertainment but in such studies much smaller samples were assessed. Overall, the literature suggests that delirium is substantially under-documented in DS and under-coded in HAS.

UK guidelines explicitly recommend using the term *delirium* in DS to support continuity of care.^5,6^ We identified several studies where descriptors or synonyms were documented rather than *delirium,* including *confusion, drowsiness, agitation*, and *disoriented*.^19,21,22,35,40^ Even when delirium is detected in practice, the diagnosis is not always documented in DS.^1,19–21,36^ Coders rely on information provided in medical records, including DS, to assign relevant administrative diagnostic codes. When delirium is missed from DS, this reduces the likelihood of delirium being captured in HAS. A further factor is coding relating to *encephalopathy* rather than *delirium*.^24,30,38,40^ We note that most studies were set in the USA, where coding practices concerning delirium are more complex and, frequently, alternative terms such as *encephalopathy* are used because of greater reimbursement.^44^ This emphasises the importance of accurate delirium documentation in DS to inform accurate delirium coding in HAS, and the need for additional training for coders.

There are several consequences of under-documentation and under-coding of delirium.^5–7^ Patients and carer partners may not know that delirium has occurred, and healthcare providers will not have an accurate past medical history.^5,7^ Patients with delirium are at higher risk of developing future dementia; screening for dementia is likely to be missed without clear communication on hospital discharge.^1,4^ We found comparatively higher documentation and coding rates in surgical services^30,36^; this may be due to more frequent and standardised perioperative observations. Findings on delirium documentation and coding were inconclusive regarding race.^18,30^However, this requires further research as there is evidence of over-diagnosis of some mental illnesses in black (and other minority ethnic) populations, and disparities in diagnostic code use.^45^

This is the first systematic review to examine the literature on delirium documentation and coding rates in DS and HAS, though there are several limitations. Though we scoped grey literature for relevant publications, we restricted our search to studies published in peer-reviewed journals. We could not explore variations in delirium documentation and coding in hyperactive and hypoactive forms of delirium, or when superimposed on dementia despite.^26,30,32,36,38^ We only looked at delirium documentation or coding rates among those who had delirium, and did not explore the specificity of delirium documentation or coding in patients without delirium. The majority of studies had moderate-to-high RoB, limiting overall conclusions.

Poor documentation of delirium stems from poor recognition of delirium. Additional research is needed to understand more about what detection methods are effective in practice, including routine use of brief delirium assessment tools that can be reliably performed at scale by non-expert staff.^46^ Further essential steps are to improve how delirium is documented in DS and coded in HAS.^22–24,27,32,40^ A multicomponent strategy involving education and training of all relevant staff (including coders) and implementing mandatory cognitive screening for delirium via electronic patient records has been shown to improve the rates of delirium detection, documentation and coding.^26,31^ Future studies should explore variables such as hospital settings, demographics and the influence of staff roles in delirium documentation and coding rates. Strategic efforts to improve delirium recognition and documentation are likely to positively affect individual patients’ quality of care and system-wide policy approaches to this common and serious condition.

## Supplementary Material

Supplementary figure

Supplementary file

## Figures and Tables

**Table 1:** Summary of included studies

Author, year of publication	Country	Study design	Type of Hospital (unit)	Mean Age (SD)^a^	Summary of study aims	Summary of study selection criteria
Alhaidari 2018	New Zealand	Retrospective review of medical records	Tertiary teaching hospital (general medicine)	N/A	To assess and potentially improve a hospital-wide delirium program.	Latest 100 general medicine patients discharged prior to 14 September 2014 with a minimal LOS of three days.
Bellelli 2015	Italy	Prospective cohort multicentre study	Acute hospitals (medical wards)	79.1 (7.3)	To describe the prevalence and impact on in-hospital mortality of delirium identified through ICD-9 codes.	Adults aged ≥65 years who underwent SBT assessment within 72 hours of admission.
Bui 2017	United States	Retrospective cohort study	Tertiary academic medical centre (surgical ICU)	61.0 (16.0)	To compare the proportions of surgical ICU patients with delirium detected using CAM-ICU who received administrative delirium documentation.	Adults aged ≥18 years admitted to surgical ICU from 1 June 2012 to 31 May 2013.
Campbell 2014	United States	Secondary data analysis from an RCT	Public hospital (general medical ward)	Overall: N/A	To evaluate the influence of race in the screening and documentation of delirium.	Adults aged ≥65 years admitted to a general medical ward of Eskenazi Hospital who spoke English.
				African American: 78.6 (8.3)		
				Non-African American: 75.3 (7.4)		
Casey 2019	Australia	Cross-sectional point prevalence survey	Australian metropolitan public health service consisting of 5 hospitals	73.0 (16.4)	To determine the extent to which ICD codes represent delirium occurrence.	Adults aged ≥18 years admitted as overnight stay on medical, surgical, specialist medicine, rehabilitation, or palliative care wards.
Chuen 2021	Canada	Retrospective chart review	Academic tertiary acute care Hospital (medical and surgical)	79.6 (8.4)	To determine the frequency and quality of delirium documentation in DS.	Adults aged ≥65 years admitted to any one of 3 academic tertiary acute care hospitals by a medical or surgical service between 1 April and 30 June 2016.
Detweiler 2014	United States	Retrospective review of medical records	Veterans medical centre (ED, medicine, surgery, psychiatry and consult liaison)	70.0 (12.9)	To assess the prevalence of missed delirium in acute care veterans coded as not having a diagnosis of delirium.	Inpatient cases of veterans that had not been coded at admission and/or discharge as having delirium
Glick 1996	United States	Retrospective chart review	General hospital (N/A)	63.8 (N/A)	To determine whether diagnosis and treatment of delirium in IABP-treated patients correlates with delirium recording at discharge.	IABP placement at the Massachusetts General Hospital in 1988.
Heriot 2017	Australia	Retrospective study	Large metropolitan private hospital (CICM)	N/A	To compare incidences of delirium in elderly intensive care patients.	Participants drawn from a larger 24 month QoL follow-up study in patients aged?80 years following ICU admission.
Hope 2014	United States	Stimulated reporting design and chart review	VA medical facility (acute medicine, surgery, neurology and ICU)	Documented delirium: 68.4 (12.0)	To assess how confirmed cases of delirium are documented in EHR.	Admitted patients with bedside diagnosis of delirium between 1 December 2009 and 31 May 2010.
				Undocumented delirium: 71.0 (12.2)		
Inouye 2005	United States	Prospective validation study	Urban teaching hospital (general medicine)	80.0 (6.5)	To validate a chart-based method for identification of delirium and compare it with direct interviewer assessment.	Patients aged ≥70 years with no delirium on admission, but at least intermediate risk for delirium at baseline.
Johnson 1992	United States	Prospective observational design and retrospective record review	University hospital (non-critical care medical unit)	N/A	To determine the sensitivity of using alternative retrospective approaches for diagnosing delirium.	Medically ill patients aged >70 years admitted between Sunday afternoons and Friday evenings who were not patient transfers, terminally ill, not admitted on weekends or for short-stays.
Kales 2003	United States	Retrospective study	VA medical facility	72.0 (7.4)	To determine the rate of recorded delirium.	Veterans aged ≥60 years at discharge with ICD-9CM code from VA.
Katznelson 2010	Canada	Prospective and retrospective study	General hospital (ICU)	63.0 (13.0)	To determine the incidence of delirium after cardiac surgery.	Cardiac surgical patients.
Kelly 2012	United States	Retrospective chart review	Tertiary referral hospital (surgery, oncology, neurology, PICU, general paediatrics, haematology, cardiology and pulmonology)	N/A	To identify the frequency of recognised and documented delirium at discharge.	Discharged patients between January 2003 and January 2011
McCoy 2017	United States	N/A	Academic medical centres	57.0 (18.7)	To characterise incidence of recorded delirium across 2 major health centres.	Inpatients aged ≥18 years with documented discharge from non-obstetrical care between 2005 and 2013.
Pendlebury 2020	United Kingdom	Prospective observational study	General hospital (acute general medicine)	70.0 (19.2)	To determine the impact of the multicomponent intervention on hospital administrative coding for delirium.	Consecutive unselected admissions to one acute medicine team over five 8-week cycles.
Ruangratsamee 2016	Thailand	Prospective and retrospective patient evaluation	Tertiary referral hospital (geriatric medicine)	78.6 (5.9)	To investigate the rate of under-recognised delirium and explore the effect of unrecognised delirium on patient mortality.	Adults aged ≥70 years admitted to general medicine between January and March 2009.
Sanchez 2013	Colombia	Cross-sectional study	Tertiary hospital (acute medicine)	N/A	To clarify the state of delirium diagnosis and records in a tertiary level public hospital in the city of Pereira.	Hospitalised adults aged >60 years.
Smulter 2019	Sweden	Retrospective observational analysis	University hospital (cardiothoracic surgery)	N/A	To analyse POD in clinical practice after cardiac surgery.	Adults aged ≥70 years scheduled for routine cardiac surgery with the use of cardiopulmonary bypass.
van Zyl 2003	Canada	Chart review	General teaching hospital (psychiatry)	73.3 (13.8)	To investigate prevalence of delirium reporting in DS.	Referrals to a consultation-liaison psychiatry service in a university teaching general hospital between July 2000 and September 2001.
Welch 2018	United Kingdom	Prospective cohort study	Tertiary university teaching hospital (acute admissions)	84.4 (N/A)	To assess if ongoing delirium research activity within an acute admissions unit impacts on prevalent delirium recognition.	Patients aged ≥70 years diagnosed with delirium.
Welch 2019	United Kingdom	Prospective observational study	Acute care trusts (acute medicine, geriatric medicine, other medicine, stroke, general, orthopaedic surgery and other surgery)	80.0 (8.3)	To ascertain the point prevalence of delirium across UK hospitals and the relationship to adverse outcomes.	Hospitalised adults aged ≥65 years, admitted between 12 March 2018 and 14th March 2018.
Zalon 2017	United States	Retrospective chart review	Community hospital	N/A	To analyse delirium documentation for hospitalised older adults.	Hospitalised patients aged ≥71 years, with known delirium who were enrolled in HELP at a community hospital.

a Mean age is provided where reported. SD = standard deviation.

Table abbreviations - CAM-ICU: Confusion Assessment Method (Intensive Care Unit), CICM: College of Intensive Care Medicine, ED: Emergency Department, EHR: Electronic Health Record, HELP: Hospital Elder Life Program, IABP: Intra-Aortic Balloon Pump, ICD-(9, 9CM): International Classification of Diseases (9th Revision, 9th Revision Clinical Modification), ICU: Intensive Care Unit, LOS: Length of Stay, PICU: Paediatric Intensive Care Unit, POD: Post-Operative Delirium, RCT: Randomised Controlled Trial, UK: United Kingdom, VA: Veterans Affairs.

**Table 2:** Documentation and coding rates in studies with and without additional delirium ascertainment methods

Author, year of publication	RoB rating^a^	Sample size (female %)	No. of patients with ascertained delirium (prevalence rate %)^b^	No. of Patients with delirium in DS (%)^c^	No. of patients with delirium in HAS (%)^d^	Delirium ascertainment method	Hospital coding format
Alhaidari 2017	M	100 (46.0)	49/100 (49.0)	19/49 (38.8)	19/39 (48.7)^e^	Documented features sufficient to fulfil short CAM	ICD-10
Bui 2017	M	1055 (51.0)	423/1055 (40.1)	N/A	22/423 (5.2)	CAM-ICU	ICD-9-CM
Campbell 2014	L	424(N/A)	163/424 (38.4)	N/A	52/163 (31.9)	CAM	ICD-9
Casey 2019	H	559 (54.6)	91/559 (16.3)	N/A	Overall: 58/559 (10.3) Study-ascertained delirium: 31/91 (34.1)	4AT 3D-CAM	ICD-10
Chuen2021	H	110 (44.5)	110/110 (100.0)	70/110 (63.6)	N/A	CHART-DEL	N/A
Detweiler 2014	H	183 (3.3)	52/183 (28.4)	5/52 (9.6)	N/A	DSM-IV TR	N/A
Glick 1996	H	Overall: 195 (N/A) Sub-study: 67 (N/A)^f^	67/195 (34.4)	Overall: 12/195 (6.2) Sub-study: 8/67 (11.9)^f^	N/A	DSM-III	N/A
Heriot 2017	M	348 (41.9)	104/348 (29.9)	N/A	36/104 (34.6)	DSM-IV Chart review	ICD-10
Hope 2014	H	25^g^ (4.0)	25/25^g^ (100.0)	11/25 (44.0)	7/25 (28.0)	DMHC notes Chart review	ICD-9
Inouye 2005	H	919 (60.0)	115/919 (12.5)	N/A	3/115 (2.6)	CAM MMSE	ICD-9CM
Johnson 1992	H	235 (N/A)	48/235 (20.4)	N/A	2/47^h^ (4.3)	MMSE BPRS DSM-III Clinical/Psychiatric examination	ICD-9CM
Katznelson 2010	M	1528 (29.0)	182/1528 (11.8)	N/A	46/182 (25.3)	CAM-ICU	ICD-10
Pendlebury 2020	M	1281 (52.0)	320/1281 (25.0)	N/A	111/320 (34.7)^i^	DSM-IV	ICD-10l^i^
Ruangratsamee 2016	M	225 (59.1)	110/225 (48.9)	16/110 (14.5)	N/A	DSM-IV	N/A
Sanchez 2013	H	5325 (N/A)	410/5325 (7.7)	N/A	N/A (29.5)	DSM-IV	ICD-10
Smulter 2019	M	142 (30.8)	78/142 (54.9)	41/78 (52.6)	16/78 (20.5)	OBS Scale MMSE DSM-IV-TR	ICD-10
van Zyl 2003	H	31 (64.5)	31/31 (100.0)	5/31 (16.1)	N/A	DSM-IV DRS DRS-R-98	N/A
Welch 2018	M	1327 (62.0)	125/1327 (9.4)	61/125 (49.0)	N/A	DSM-IV	N/A
Welch 2019	H	1507 (54.2)	222/1507 (14.7)	44/154 (28.6)^j^	N/A	4AT DSM-V	N/A
Zalon 2017	H	34 (82.4)	34/34 (100.0)	1/34 (2.9)	13/34 (38.2)	CAM	ICD-9
*Studies which did not use additional delirium ascertainment methods*							
Bellelli 2015	L	2521 (50.8)	N/A	N/A	72/2521 (2.9)	N/A	ICD-9
Kales 2003	H	267947 (2.0)	N/A	N/A	3978/267947 (1.5)^m^	N/A	ICD-9CM
Kelly 2012	H	Overall: 64046 (44.0) Sub-study: 53 (N/A)^l^	N/A	Overall: 89/64046 (0.1)^k^ Sub-study: 8/53 (15.1)	N/A	N/A	‘Delirium’ or ‘encephalopathy’ in ‘discharge problem list’
McCoy 2017	H	809512 (54.8)	N/A	7579/809512 (0.9)^k^	27513/809512 (3.4)^m^	N/A	ICD-9

a RoB = Risk of Bias. RoB was assessed using the EPHPP tool. In this table, we provide the Global RoB rating.

b Number of patients with study-ascertained delirium is provided in relation to the overall sample size. We assessed study prevalence rate for delirium as the number of patients with delirium (cases), as assessed by the study delirium ascertainment method, divided by the overall sample size * 100.

c Number of patients with delirium in discharge summary in relation to study-ascertained delirium N (%) and/or in relation to whole study sample if different.

d Number of patients with delirium in hospital administrative databases in relation to study-ascertained delirium N (%) and/or in relation to whole study sample if different.

e The authors reported ICD-9 coding rates in 39 of the 49 patients with delirium documented in clinical records.

f The authors reported on a sub-group of patients who had diagnosis of delirium made by a retrospective chart review.

g The authors reported documentation and coding rates in reference to the overall sample size (N=25).

h The authors reported patient records for 47 of the 48 patients with delirium were available.

i Though the authors reported an overall coding rate of 34.7% in HAS, there was a big increase over time in coding rates from 12.8% in 2010 to 60.2% in 2018.

j The authors reported that discharge documentation were available for 154 of the 222 patients identified with study-ascertained delirium.

k Number of patients with delirium in DS in relation to whole study sample N(%).

l The authors also reported on a sub-group of patients who had a diagnosis of delirium previously made by the clinical team.

m Number of patients with delirium in HAS in relation to whole study sample N(%).

Data not given in published study or where data is not applicable is denoted as “N/A” (not available/applicable).

All values rounded to 1 decimal place.

Table abbreviations - 3D-CAM: 3 Minute Diagnostic Assessment using Confusion Assessment Method, 4AT: The 4 ‘A’s Test, BPRS: Brief Psychiatric Rating Scale, CAM-(ICU): Confusion Assessment Method (Intensive Care Unit), CHART-DEL: Chart-based Delirium Identification Instrument, DMHC: Delirium Mental Health Consult, DRS: Delirium Rating Scale, DRS-R-98: Delirium Rating Scale Revised, DSM (III, IV, IV-TR, V): Diagnostic and Statistical Manual of Mental Disorders (3rd Edition, 4th Edition, 4th Edition-Text Revision, 5th Edition), ICD- (9, 9CM, 10): International Classification of Diseases (9th Revision, 9th Revision Clinical Modification, 10th Revision), MMSE: Mini-Mental State Examination, OBS Scale: Organic Brain Syndrome Scale.
