# Supplementary figures and images for: Delirium is under-reported in discharge summaries and in hospital administrative systems: a systematic review
Source: Delirium (Bielef). Author manuscript; Available in PMC 2024 Dec 9. (PMC7617113; doi:10.56392/001c.74541)

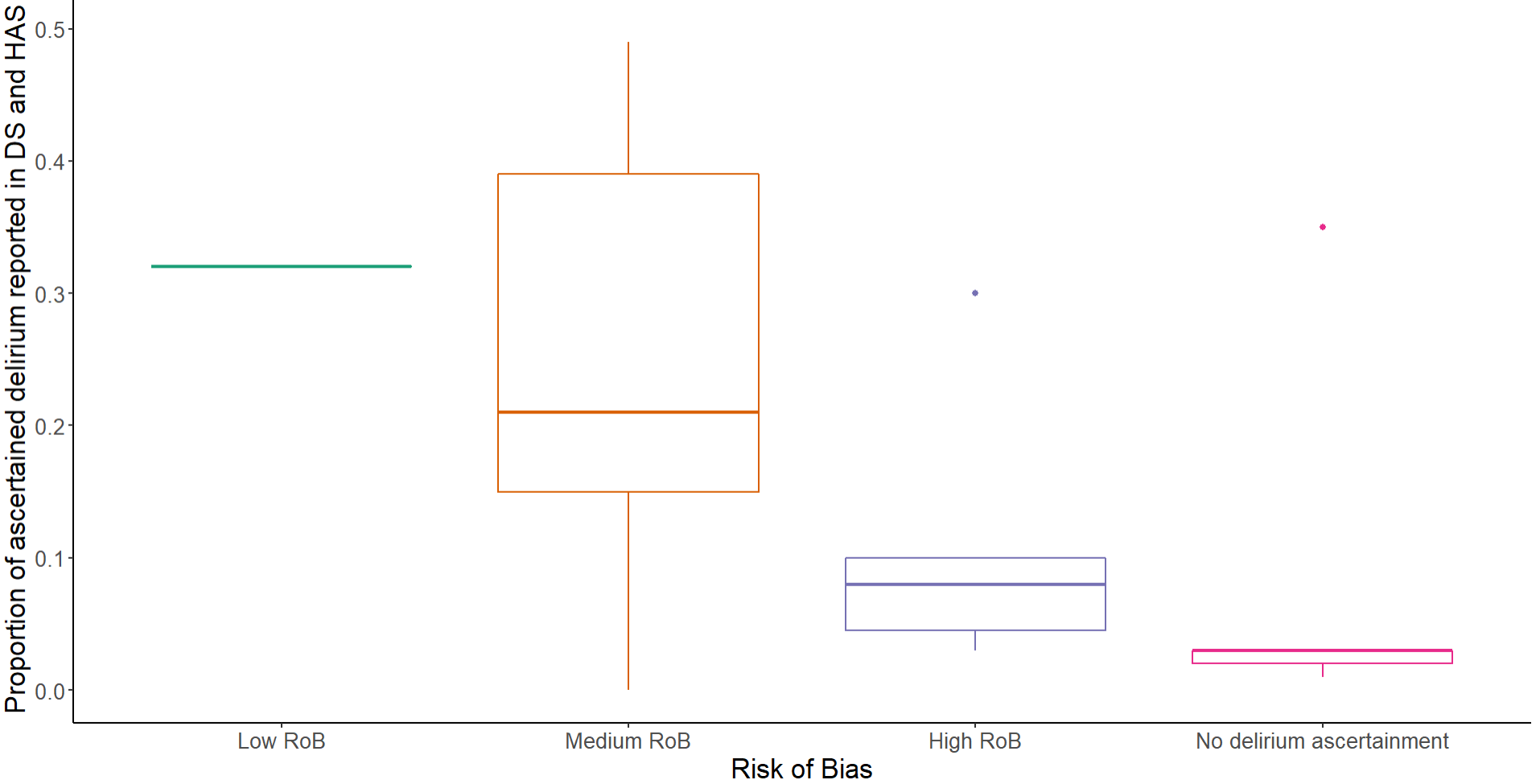

Supplement: Supplementary figure [file EMS176155-supplement-Supplementary_figure.png]
