# Supplementary material for: Delirium is under-reported in discharge summaries and in hospital administrative systems: a systematic review
Source: Delirium (Bielef). Author manuscript; Available in PMC 2024 Dec 9. (PMC7617113; doi:10.56392/001c.74541)
Supplement: Supplementary file [file EMS176155-supplement-Supplementary_file.docx]

**Full Methods**

The systematic review was registered with PROSPERO on 26 February 2021 (CRD42021239547) and is reported according to PRISMA guidelines (supplementary Table 1, supplementary Figure 1).^15^

**Inclusion criteria**

Studies using any peer-reviewed study methodology which had:

- all, or a proportion of, patients with delirium in a hospital setting – including specific subtype of delirium, or delirium superimposed on dementia, and
- documented description and/or diagnosis of delirium in DS (or equivalent), or
- description of HAS coding used to record delirium diagnosis, and
- publication in English or translatable to English using translation tools.

No restrictions were placed on demographic variables such as age, ethnicity and sex.

**Exclusion criteria**

To minimise bias, studies were excluded if they:

- did not include delirium as a diagnosis (e.g. used only synonyms such as ‘confusion’ or ‘encephalopathy’ or ‘acute psychosis’ or ‘altered mental status’) or used only symptoms of delirium, or
- did not specifically refer to DS (or equivalent) to report on delirium documentation rates, or
- reported delirium in non-hospital settings, such as care homes and hospices, or
- were systematic reviews, meta-analyses, abstracts, letters to editors or opinion pieces.

**Search strategy**

We sought advice on developing a search strategy from an academic librarian at the University of Edinburgh. The search strategy comprised three concepts: (1) delirium, (2) documentation or coding, and (3) DS or HAS. Search strategies were developed for Medline, Embase, PsycINFO and Web of Science (Supplementary Table 2), searching Embase and Medline from inception (1980 and 1966, respectively) and other databases from 1949 when delirium was first coded in the International Statistical Classification of Diseases.^16^

The search was performed on 13 March 2021 and updated on 23 June 2021. We used the forward citation technique on eligible studies to identify any relevant peer-reviewed publications. We also scoped grey literature using the same concepts (Supplementary Table 3). Title, abstract and keyword screening, and full-text reviews of long-listed publications, were performed independently by two reviewers (TI and SS). Conflicts were resolved by an additional reviewer (AMJM).

**Specific Sub-groups**

To explore the variations in delirium documentation and/or coding rates, we also extracted data from studies that additionally reported rates based on:

- different sub-groups of the population (e.g. race or gender),
- different hospital settings (e.g. geriatrics, medical, or intensive care units),
- structured and unstructured DS,
- different hospital staff (e.g. physicians or nurses).

**Risk of bias**

Two reviewers (TI and SS) independently assessed studies for risk of bias (RoB) using the Effective Public Health Practice Project (EPHPP) quality assessment tool.^17^ Conflicts were resolved through discussion. Studies were assessed as strong, moderate or weak, across different methodological areas: selection bias, study design, confounding, blinding and data collection (Supplementary Table 4). We applied the global rating criteria for an overall rating. Global ratings for RoB generally ranged from moderate to high, largely due to study design, confounders and blinding (Figure 1). Two studies had low global RoB ratings.^18,19^

**Data extraction and synthesis measures**

For each study we extracted the reported delirium documentation and/or coding rates in DS and HAS, respectively. Where studies used a range of codes to denote presumed delirium or synonyms (e.g. encephalopathy) but reported rates by specific code, we calculated the coding rates by delirium-specific codes only (Supplementary Table 5). Similarly, where studies did not use a diagnostic manual or coding dictionary, but instead used text in the DS, we reported the documentation rates only for the specific term ‘delirium’ rather than synonyms.

Some studies measured delirium with additional study-specific ascertainment methods, for example through chart reviews for evidence of delirium. For these we calculated *study-prevalence* rates for delirium by dividing the total number of cases (n), as determined by the delirium study-ascertainment method used, by the sample size in which delirium had been ascertained by the study (N) (Supplementary Table 6). Amongst patients with study-ascertained delirium, we extracted the reported number and proportion of patients with either delirium documentation in DS or HAS clinical code for delirium. Where a study did not report the proportion, we calculated this manually.

Supplementary Figure 1. **PRISMA Flow Diagram**

Supplementary Table 1. **PRISMA Checklist**

| **Section and Topic** | **Item #** | **Checklist item** | **Location where item is reported** |
| --- | --- | --- | --- |
| **TITLE** | | |  |
| Title | 1 | Identify the report as a systematic review. | 1 |
| **ABSTRACT** | | |  |
| Abstract | 2 | See the PRISMA 2020 for Abstracts checklist. | 2 |
| **INTRODUCTION** | | |  |
| Rationale | 3 | Describe the rationale for the review in the context of existing knowledge. | 3 |
| Objectives | 4 | Provide an explicit statement of the objective(s) or question(s) the review addresses. | 4 |
| **METHODS** | | |  |
| Eligibility criteria | 5 | Specify the inclusion and exclusion criteria for the review and how studies were grouped for the syntheses. | 5 |
| Information sources | 6 | Specify all databases, registers, websites, organisations, reference lists and other sources searched or consulted to identify studies. Specify the date when each source was last searched or consulted. | 6, supplementary table 2 |
| Search strategy | 7 | Present the full search strategies for all databases, registers and websites, including any filters and limits used. | 7 and supplementary table 3 |
| Selection process | 8 | Specify the methods used to decide whether a study met the inclusion criteria of the review, including how many reviewers screened each record and each report retrieved, whether they worked independently, and if applicable, details of automation tools used in the process. | 8 |
| Data collection process | 9 | Specify the methods used to collect data from reports, including how many reviewers collected data from each report, whether they worked independently, any processes for obtaining or confirming data from study investigators, and if applicable, details of automation tools used in the process. | 9 |
| Data items | 10a | List and define all outcomes for which data were sought. Specify whether all results that were compatible with each outcome domain in each study were sought (e.g. for all measures, time points, analyses), and if not, the methods used to decide which results to collect. | 10 |
|  | 10b | List and define all other variables for which data were sought (e.g. participant and intervention characteristics, funding sources). Describe any assumptions made about any missing or unclear information. | 10 |
| Study risk of bias assessment | 11 | Specify the methods used to assess risk of bias in the included studies, including details of the tool(s) used, how many reviewers assessed each study and whether they worked independently, and if applicable, details of automation tools used in the process. | 11, supplementary table 4 |
| Effect measures | 12 | Specify for each outcome the effect measure(s) (e.g. risk ratio, mean difference) used in the synthesis or presentation of results. | Table 2 |
| Synthesis methods | 13a | Describe the processes used to decide which studies were eligible for each synthesis (e.g. tabulating the study intervention characteristics and comparing against the planned groups for each synthesis (item #5)). | 13a, Table 1 & 2 |
|  | 13b | Describe any methods required to prepare the data for presentation or synthesis, such as handling of missing summary statistics, or data conversions. | 13b, Table 1 & 2 |
|  | 13c | Describe any methods used to tabulate or visually display results of individual studies and syntheses. | 13c, Table 1 & 2 |
|  | 13d | Describe any methods used to synthesize results and provide a rationale for the choice(s). If meta-analysis was performed, describe the model(s), method(s) to identify the presence and extent of statistical heterogeneity, and software package(s) used. | 13d |
|  | 13e | Describe any methods used to explore possible causes of heterogeneity among study results (e.g. subgroup analysis, meta-regression). | N/A – systematic review and narrative synthesis |
|  | 13f | Describe any sensitivity analyses conducted to assess robustness of the synthesized results. | N/A – systematic review and narrative synthesis |
| Reporting bias assessment | 14 | Describe any methods used to assess risk of bias due to missing results in a synthesis (arising from reporting biases). | 8, Supplementary table 4, table 2 |
| Certainty assessment | 15 | Describe any methods used to assess certainty (or confidence) in the body of evidence for an outcome. | 15 |
| **RESULTS** | | |  |
| Study selection | 16a | Describe the results of the search and selection process, from the number of records identified in the search to the number of studies included in the review, ideally using a flow diagram. | 8, Figure 1 |
|  | 16b | Cite studies that might appear to meet the inclusion criteria, but which were excluded, and explain why they were excluded. | Figure 1 |
| Study characteristics | 17 | Cite each included study and present its characteristics. | 17, Table 1 |
| Risk of bias in studies | 18 | Present assessments of risk of bias for each included study. | 18, Supplementary table 4 |
| Results of individual studies | 19 | For all outcomes, present, for each study: (a) summary statistics for each group (where appropriate) and (b) an effect estimate and its precision (e.g. confidence/credible interval), ideally using structured tables or plots. | 19, Table 2 |
| Results of syntheses | 20a | For each synthesis, briefly summarise the characteristics and risk of bias among contributing studies. | 19, Table 2, Supplementary table 4 |
|  | 20b | Present results of all statistical syntheses conducted. If meta-analysis was done, present for each the summary estimate and its precision (e.g. confidence/credible interval) and measures of statistical heterogeneity. If comparing groups, describe the direction of the effect. | 20b |
|  | 20c | Present results of all investigations of possible causes of heterogeneity among study results. | N/A – systematic review and narrative synthesis |
|  | 20d | Present results of all sensitivity analyses conducted to assess the robustness of the synthesized results. | N/A – systematic review and narrative synthesis |
| Reporting biases | 21 | Present assessments of risk of bias due to missing results (arising from reporting biases) for each synthesis assessed. | 19, 23a, 23b Table 2, Supplementary table 4 |
| Certainty of evidence | 22 | Present assessments of certainty (or confidence) in the body of evidence for each outcome assessed. | 19, 23a, 23b Table 2, Supplementary table 4 |
| **DISCUSSION** | | |  |
| Discussion | 23a | Provide a general interpretation of the results in the context of other evidence. | 23a |
|  | 23b | Discuss any limitations of the evidence included in the review. | 23b |
|  | 23c | Discuss any limitations of the review processes used. | 23c |
|  | 23d | Discuss implications of the results for practice, policy, and future research. | 23d |
| **OTHER INFORMATION** | | |  |
| Registration and protocol | 24a | Provide registration information for the review, including register name and registration number, or state that the review was not registered. | 24a |
|  | 24b | Indicate where the review protocol can be accessed, or state that a protocol was not prepared. | 24b |
|  | 24c | Describe and explain any amendments to information provided at registration or in the protocol. | 24c |
| Support | 25 | Describe sources of financial or non-financial support for the review, and the role of the funders or sponsors in the review. | 26 |
| Competing interests | 26 | Declare any competing interests of review authors. | 26 |
| Availability of data, code and other materials | 27 | Report which of the following are publicly available and where they can be found: template data collection forms; data extracted from included studies; data used for all analyses; analytic code; any other materials used in the review. | 24a, 24b, Supplementary table 2 |

*From:*  Page MJ, McKenzie JE, Bossuyt PM, Boutron I, Hoffmann TC, Mulrow CD, et al. The PRISMA 2020 statement: an updated guideline for reporting systematic reviews. BMJ 2021;372:n71. doi: 10.1136/bmj.n71

For more information, visit: <http://www.prisma-statement.org/>

Supplementary Table 2. **Search Algorithms**

| **EMBASE:**  **Name of Database**: Ovid Embase  **Time Span**: 1980 to 23-Jun-2021  **Results**: 4028  **Search algorithm**:   1. delirium/ 2. deliri*.ti,ab 3. **OR/1-2** 4. exp hospital discharge 5. (discharge adj (code* OR coding OR letter* OR document* OR case* OR record* OR summar* OR note$1 OR diagnos*)).mp. 6. Documentation.ti,ab. 7. exp medical record 8. exp coding 9. exp coding algorithm 10. ICD*.mp. 11. ((administrati*) adj (claim* OR code* OR coding)).mp. 12. (unrecogni* OR unreport*). ti,ab. 13. (diagnos* adj (code* OR coding OR accura* )). ti,ab. 14. (under adj (diagnos* OR record* OR report* OR coding)). ti,ab. 15. **OR/4-14** 16. **AND/3,15** | **MEDLINE:**  **Name of Database**: Ovid Medline  **Time Span**: 1966 to 23-Jun-2021  **Results**: 870  **Search algorithm**:   1. delirium/ 2. deliri*.ti,ab. 3. **OR/1-2** 4. (unrecogni* OR unreport*). ti,ab. 5. (diagnos* adj (code* OR coding OR accura* )). ti,ab. 6. (under adj (diagnos* OR record* OR report* OR coding)). ti,ab. 7. (discharge adj (code* OR coding OR letter* OR document* OR case* OR record* OR summar* OR note$1 OR diagnos*)).mp. 8. ICD*.mp. 9. ((administrati*) adj (claim* OR code* OR coding)).mp. 10. (Clinical OR Hospital) adj (code* OR coding OR report*).mp. 11. algorithms.ti,ab 12. Documentation.ti,ab. 13. exp medical records 14. exp Nursing Records 15. exp Hospital Records 16. exp Clinical Coding 17. **OR/4-16** 18. **AND/3,17** | **PSYCINFO:**  **Name of Database**: Ovid PsycINFO  **Time Span**: 1949 to 23-Jun-2021  **Results**: 344  **Search algorithm**:   1. exp Delirium/ 2. deliri*.ti,ab. 3. **OR/1-2** 4. (unrecogni* OR unreport*).ti,ab. 5. (diagnos* adj (code* OR coding OR accura* )). ti,ab. 6. (under adj (diagnos* OR record* OR report* OR coding)). ti,ab. 7. (discharge adj (code* OR coding OR letter* OR document* OR case* OR record* OR summar* OR note$1 OR diagnos*)).mp. 8. ((administrati*) adj (claim* OR code* OR coding)).mp. 9. algorithms.ti,ab 10. Documentation.ti,ab. 11. (Clinical OR Hospital) adj (code* OR coding OR report*).mp. 12. ICD*.mp. 13. exp medical records 14. exp facility discharge 15. exp institutional release 16. **OR/4-15** 17. **AND/3,16** | **WEB OF SCIENCE:**  **Name of Database**: Web of science  **Time Span**: 1949 to 23-Jun-2021  **Results**: 2667  **Search algorithm**:   1. ALL=delirium 2. TS=deliri* 3. **OR/1-2** 4. TS=(discharge near/2 (document* OR record* OR note* OR report* OR diagnos* OR code* OR coding OR summar*)) 5. TS=(diagnostic near/2 (code* OR coding OR accura*)) 6. TS=documentation 7. TS=misdiagnos* 8. TS=(under near/2 (coding OR record* OR report* OR diagnos*)) 9. TS =((hospital OR medical OR facility OR institution*) AND (discharge)) 10. TS=((clinical) AND (coding)) 11. TS=((administrati*) AND (claim* OR code* OR coding)) 12. TS=((code* OR coding) near/2 (algorithm)) 13. TS=ICD 14. TS=DSM 15. ALL="medical record" 16. ALL=("electronic health record" OR EHR) 17. **OR/4-16** 18. **AND/3,17** |
| --- | --- | --- | --- |

*The search strategy was generated by developing keywords for each concept and further tested and refined by implementing truncation and wild card methods to narrow the number of results yielded.*

Supplementary Table 3. **Societies and Associations scoped**

| **Delirium Societies** | **Geriatric Societies** | **Acute Medicine Societies** |
| --- | --- | --- |
| Scottish Delirium Association, American Delirium Society, European Delirium Association, Australasian Delirium Association | British Geriatrics Society, European Geriatric Society | Society of Acute Medicine, Royal College of Emergency Medicine |

Supplementary Table 4. **Risk of bias using EPHPP Assessment Tool**

| Author, Year | Selection bias | Study design | Confounders | Blinding | Data collection methods | Withdrawals  and  drop-outs* | Global rating for paper |
| --- | --- | --- | --- | --- | --- | --- | --- |
| Alhaidari et al, 2017 | L | M | H | M | L |  | M |
| Bellelli et al, 2015 | L | M | M | M | L |  | L |
| Bui et al, 2017 | M | M | M | H | L |  | M |
| Campbell et al, 2014 | M | M | M | L | L |  | L |
| Casey et al, 2019 | L | H | M | H | L |  | H |
| Chuen et al, 2021 | M | H | M | H | L |  | H |
| Detweiler et al, 2014 | H | M | H | H | L |  | H |
| Glick et al, 1996 | H | H | H | H | L |  | H |
| Heriot et al, 2017 | M | M | M | H | L |  | M |
| Hope et al, 2014 | L | H | H | M | L |  | H |
| Inouye et al, 2005 | L | H | M | H | L |  | H |
| Johnson et al, 1992 | H | M | H | M | L |  | H |
| Kales et al, 2003 | M | H | H | H | L |  | H |
| Katznelson et al, 2010 | M | M | H | M | L |  | M |
| Kelly et al, 2012 | M | M | H | H | L |  | H |
| McCoy et al, 2017 | L | H | H | U | L |  | H |
| Pendlebury et al, 2020 | L | M | M | H | L |  | M |
| Ruangratsamee  et al, 2016 | M | M | L | H | L |  | M |
| Sanchez et al, 2013 | M | M | H | H | L |  | H |
| Smulter et al, 2019 | M | M | H | L | L |  | M |
| van Zyl et al, 2003 | H | H | H | H | L |  | H |
| Welch et al, 2018 | L | M | H | L | L |  | M |
| Welch et al, 2019 | M | M | H | H | L |  | H |
| Zalon et al, 2017 | H | H | H | H | M |  | H |
| *Due to the methodologies used by the studies, withdrawals and drop-outs were deemed as not applicable | | | | | | | |

**KEY**

STRONG (no WEAK ratings)

MODERATE (one WEAK rating)

WEAK (two or more WEAK ratings)

| **EPHPP Rating** | **Risk of Bias Rating** |
| --- | --- |
| Strong | Low (L) |
| Moderate | Moderate (M) |
| Weak | High (H) |
| Unclear | Unclear (U) |
| N/A |  |

Supplementary Table 5. **Delirium HAS codes used by studies and their definitions**

| **ICD-9/ICD-9CM** |  |
| --- | --- |
| 290.11 | Pre-senile dementia with delirium |
| 290.3 | Senile dementia with delirium |
| 290.41 | Vascular dementia, with delirium |
| 292.0, 292.81 | Drug-induced delirium |
| 293.0 | Acute Delirium |
| 293.1 | Subacute delirium |
| **ICD-10** |  |
| F05.0 | Delirium not superimposed on dementia |
| F05.1 | Delirium superimposed on dementia |
| F05.8 | Other delirium |
| F05.9 | Delirium unspecified |

Supplementary Table 6. **Study-ascertained delirium prevalence, HAS coding and DS documentation rates**

| Author, Year^1^ | Prevalence and Rates^2^ | Estimate (95% CI)^3^ |
| --- | --- | --- |
| Alhaidari, 2017 | Study-ascertained delirium prevalence | 0.49 (0.39, 0.59) |
| Alhaidari, 2017 | DS delirium documentation rate | 0.19 (0.13, 0.28) |
| Alhaidari, 2017 | HAS delirium coding rate | 0.19 (0.13, 0.28) |
| Bellelli, 2015 | HAS delirium coding rate | 0.03 (0.02, 0.04) |
| Bui, 2017 | Study-ascertained delirium prevalence | 0.40 (0.37, 0.43) |
| Bui, 2017 | HAS delirium coding rate | 0.02 (0.01, 0.03) |
| Campbell, 2014 | Study-ascertained delirium prevalence | 0.38 (0.34, 0.43) |
| Campbell, 2014 | HAS delirium coding rate | 0.12 (0.09, 0.16) |
| Casey, 2019 | Study-ascertained delirium prevalence | 0.16 (0.13, 0.20) |
| Casey, 2019 | HAS delirium coding rate | 0.06 (0.04, 0.08) |
| Detweiler, 2014 | Study-ascertained delirium prevalence | 0.28 (0.22, 0.35) |
| Detweiler, 2014 | DS delirium documentation rate | 0.03 (0.01, 0.06) |
| Glick, 1996 | Study-ascertained delirium prevalence | 0.34 (0.28, 0.41) |
| Glick, 1996 | DS delirium documentation rate | 0.04 (0.02, 0.08) |
| Heriot, 2017 | Study-ascertained delirium prevalence | 0.30 (0.25, 0.35) |
| Heriot, 2017 | HAS delirium coding rate | 0.10 (0.08, 0.13) |
| Inouye, 2005 | Study-ascertained delirium prevalence | 0.13 (0.11, 0.15) |
| Inouye, 2005 | HAS delirium coding rate | 0.00 (0.00, 0.01) |
| Johnson, 1992 | Study-ascertained delirium prevalence | 0.20 (0.16, 0.26) |
| Johnson, 1992 | HAS delirium coding rate | 0.01 (0.00, 0.03) |
| Kales, 2003 | HAS delirium coding rate | 0.01 (0.01, 0.02) |
| Katznelson, 2010 | Study-ascertained delirium prevalence | 0.12 (0.10, 0.14) |
| Katznelson, 2010 | HAS delirium coding rate | 0.03 (0.02, 0.04) |
| Kelly, 2012 | DS delirium documentation rate | 0.00 (0.00, 0.00) |
| McCoy, 2017 | DS delirium documentation rate | 0.01 (0.01, 0.01) |
| McCoy, 2017 | HAS delirium coding rate | 0.03 (0.03, 0.03) |
| Pendlebury, 2020 | Study-ascertained delirium prevalence | 0.25 (0.23, 0.27) |
| Pendlebury, 2020 | HAS delirium coding rate | 0.09 (0.07, 0.10) |
| Ruangratsamee, 2016 | Study-ascertained delirium prevalence | 0.49 (0.42, 0.55) |
| Ruangratsamee, 2016 | DS delirium documentation rate | 0.07 (0.04, 0.11) |
| Sanchez, 2013 | Study-ascertained delirium prevalence | 0.08 (0.07, 0.08) |
| Sanchez, 2013 | HAS delirium coding rate | 0.02 (0.02, 0.03) |
| Smulter, 2019 | Study-ascertained delirium prevalence | 0.55 (0.47, 0.63) |
| Smulter, 2019 | DS delirium documentation rate | 0.29 (0.22, 0.37) |
| Smulter, 2019 | HAS delirium coding rate | 0.11 (0.07, 0.18) |
| Welch, 2018 | Study-ascertained delirium prevalence | 0.09 (0.08, 0.11) |
| Welch, 2018 | DS delirium documentation rate | 0.05 (0.04, 0.06) |

Studies that reported study-ascertained delirium prevalence from a sample *and* reported DS documentation and/or HAS coding rates were included. We excluded studies where the overall sample comprised 100% delirium patients as determined by, e.g., retrospective chart review. As a result, the following studies were excluded: Chuen et al., 2021, Hope et al., 2014, van Zyl et al., 2003 and Zalon et al., 2007. We also excluded Welch et al., 2019; the authors reported that discharge documentation were available for 154 of the 222 patients identified with study-ascertained delirium.

2 DS documentation and/or HAS coding rates are expressed as a fraction of the overall sample size.

3 95% confidence interval
